# Supplementary material for: Deep transfer learning for inter-chain contact predictions of transmembrane protein complexes
Source: Nat Commun. 2023 Aug 15;14:4935. doi: 10.1038/s41467-023-40426-3 (PMC10427616; doi:10.1038/s41467-023-40426-3)
Supplement: Supplementary file 1 — Supplementary Information [file 41467_2023_40426_MOESM1_ESM.pdf]

Supplementary Information

Deep transfer learning for inter-chain contact predictions of transmembrane protein complexes

Peicong Lin, Yumeng Yan, Huanyu Tao and Sheng-You Huang

School of Physics, Huazhong University of Science and Technology, Wuhan, Hubei 430074, P. R. China

Key Words: Protein-protein interaction, Transmembrane protein, Residue contact prediction, Transfer learning, Deep learning

Running Title: Inter-protein contact prediction for transmembrane protein complexes

Email: huangsy@hust.edu.cn; Phone: +86-27-87543881; Fax: +86-027-87556576

Supplementary Table 1: Comparison of the precisions (%) by DeepTMP, DeepHomo2.0, CDPred, GLINTER, and DeepHomo for top 1, 10, 25, 50, L/10, L/5, and L predicted contacts on the 296 soluble protein dimers from Homodimer300 test set when the experimental monomer structures (predicted monomer structures by AlphaFold2) are used as input.

Method	Top 1	Top 10	Top 25	Top 50	Top L/10	Top L/5	Top L
DeepTMP	71.3 (61.5)	71.2 (62.7)	70.5 (62.1)	70.2 (61.0)	70.9 (62.0)	70.2 (61.5)	65.1 (55.1)
DeepHomo2.0	73.3 (61.8)	71.6 (61.7)	70.5 (60.2)	68.0 (57.8)	70.3 (60.6)	68.4 (58.1)	56.3 (46.8)
CDPred	74.7 (68.6)	72.3 (67.3)	70.2 (66.4)	68.4 (64.5)	70.1 (66.7)	68.3 (64.7)	58.7 (54.7)
GLINTER	68.6 (61.5)	64.4 (57.4)	60.4 (54.8)	56.6 (51.6)	61.3 (55.2)	57.5 (52.2)	43.3 (39.5)
DeepHomo	61.1 (55.4)	57.2 (49.6)	53.8 (46.4)	50.1 (44.0)	54.6 (47.6)	51.6 (44.6)	38.8 (33.6)

Supplementary Table 2: Comparison of the precisions (%) by DeepTMP and DeepTMPα for top 1, 10, 25, 50, L/10, L/5, and L predicted contacts on the three types of topologies when the experimental monomer structures (predicted monomer structures by AlphaFold2) are used as input.

Method	Topology	Top 1	Top 10	Top 25	Top 50	Top L/10	Top L/5	Top L
DeepTMP	Alpha	76.3 (71.1)	76.6 (70.0)	74.8 (67.7)	72.5 (64.9)	76.2 (68.8)	73.7 (66.4)	62.1 (56.4)
	Beta	100 (100)	100 (96.7)	98.7 (94.7)	98.7 (90.0)	97.7 (96.6)	98.3 (90.2)	87.9 (74.3)
	Alpha+Beta	100 (90.9)	97.3 (93.6)	97.8 (93.8)	98.0 (90.9)	97.9 (92.3)	96.9 (89.0)	85.0 (79.7)
DeepTMPα	Alpha	65.8 (68.4)	70.8 (67.1)	68.7 (66.6)	67.1 (65.1)	70.8 (66.3)	68.6 (64.1)	58.2 (54.0)
	Beta	100 (100)	96.7 (96.7)	84.0 (92.0)	82.0 (87.3)	84.2 (90.8)	83.0 (87.3)	74.5 (65.7)
	Alpha+Beta	90.9 (90.9)	94.5 (89.1)	93.8 (90.2)	91.8 (88.9)	92.2 (89.4)	89.5 (88.2)	76.9 (73.1)

Supplementary Table 3: The precisions of DeepTMP on extracellular, cytoplasmic and transmembrane segments for top 1, 10, 25, 50, L/10, L/5, and L predicted contacts on the test set of 52 transmembrane protein complexes when the experimental monomer structures (predicted monomer structures by AlphaFold2) are used as input

Regions	Top 1	Top 10	Top 25	Top 50	Top L/10	Top L/5	Top L
extracellular	75.0 (66.7)	73.2 (78.0)	80.3 (71.9)	77.7 (67.0)	78.6 (71.7)	73.5 (72.1)	61.7 (57.7)
cytoplasmic	100 (90.0)	97.6 (74.7)	84.5 (73.7)	84.2 (68.7)	88.1 (71.1)	84.6 (67.0)	72.1 (60.8)
transmembrane	84.6 (83.3)	82.5 (70.9)	81.5 (72.2)	80.1 (71.6)	86.1 (74.9)	78.2 (73.2)	66.0 (60.2)
