# Supplementary material for: Deep transfer learning for inter-chain contact predictions of transmembrane protein complexes
Source: Nat Commun. 2023 Aug 15;14:4935. doi: 10.1038/s41467-023-40426-3 (PMC10427616; doi:10.1038/s41467-023-40426-3)
Supplement: Supplementary file 3 — Description of Additional Supplementary Files [file 41467_2023_40426_MOESM3_ESM.pdf]

Description of Additional Supplementary Files

File Name: Supplementary Data 1
Description:
Supplementary Data 1a: The evaluation results of DeepTMP, CDPred, DeepHomo2.0, GLINTER, DeepHomo, and DNCON2_Inter on the TMP test set of 52 transmembrane protein complexes when the experimental monomer structures are used as input. The topology for each target is also listed. "-" means no output for this test case, and top n is the number of top considered contacts, and L is the length of the sequence.
Supplementary Data 1b: The evaluation results of DeepTMP, CDPred, DeepHomo2.0, GLINTER, DeepHomo, and DNCON2_Inter on the TMP test set of 52 transmembrane protein complexes when the AlphaFold2-predicted monomer structures are used as input. The topology for each target is also listed. "-" means no output for this test case, and top n is the number of top considered contacts, and L is the length of the sequence.

File Name: Supplementary Data 2
Description:
Supplementary Data 2a: Comparison of the precisions for initial training model (IT-Model), direct training model (DT-Model), and DeepTMP when the experimental monomer structures are used as input. The number of chains and the ratio of transmembrane region for each target are also listed. Top n is the number of top considered contacts and L is the length of the sequence.
Supplementary Data 2b: Comparison of the precisions for initial training model (IT-Model), direct training model (DT-Model), and DeepTMP when the AlphaFold2-predicted monomer structures are used as input. The number of chains and the ratio of transmembrane region for each target are also listed. Top n is the number of top considered contacts and L is the length of the sequence.

File Name: Supplementary Data 3
Description:
Supplementary Data 3a: The evaluation results of DeepTMP, CDPred, DeepHomo2.0, GLINTER, and DeepHomo on the test set of 296 soluble protein complexes when the experimental monomer structures are used as input. Top n is the number of top considered contacts and L is the length of the sequence.
Supplementary Data 3b: The evaluation results of DeepTMP, CDPred, DeepHomo2.0, GLINTER, and DeepHomo on the test set of 296 soluble protein complexes when the AlphaFold2-predicted monomer structures are used as input. Top n is the number of top considered contacts and L is the length of the sequence.

File Name: Supplementary Data 4
Description:
Supplementary Data 4a: The top L precisions of DeepTMP, CDPred, DeepHomo2.0, GLINTER, DeepHomo, and DNCON2_Inter versus the topology, ln(Meff), ln(Meff) for ESM-MSA-1b, and contact density for each target on the TMP test set. "-" means no output for this test case. Top n is the number of top considered contacts, and L is the length of sequence.
Supplementary Data 4b: The precisions of DeepTMP using the MSAs from Uniref and BFD databases and the combined MSA from two databases (Uniref+BFD) when the experimental monomer structures are used as input. Top n is the number of top considered contacts, and L is the length of the sequence.

File Name: Supplementary Data 5

Description:
Supplementary Data 5a: The precisions of DeepTMP, DeepHomo2_TMP, IT-Model, and DeepHomo2.0 on the TMP test set when the experimental monomer structures are used as input. Top n is the number of top considered contacts and L is the length of the sequence.
Supplementary Data 5b: The precisions of DeepTMP, DeepHomo2_TMP, IT-Model, and DeepHomo2.0 on the TMP test set when the AlphaFold2-predicted monomer structures are used as input. Top n is the number of top considered contacts and L is the length of the sequence.

File Name: Supplementary Data 6
Description:
Supplementary Data 6a: The evaluation results of DeepTMP and DeepTMPα on the TMP test set of 52 transmembrane protein complexes when the experimental monomer structures are used as input. The topology of each target is also listed. Top n is the number of top considered contacts, and L is the length of the sequence.
Supplementary Data 6a: The evaluation results of DeepTMP and DeepTMPα on the TMP test set of 52 transmembrane protein complexes when the AlphaFold2-predicted monomer structures are used as input. The topology of each target is also listed. Top n is the number of top considered contacts, and L is the length of the sequence.

File Name: Supplementary Data 7
Description: The top 10, 25, L/5, and L precisions of DeepTMP versus the topology and average entropy of each target on the TMP test set when the experimental monomer structures are used as input. Top n is the number of top considered contacts, and L is the length of the sequence.

File Name: Supplementary Data 8
Description:
Supplementary Data 8a: The precisions of DeepTMP for extracelluar (Extra), cytopliasmic (Cyto), and transmembrane (TM) regions when the experimental monomer structures are used as input. Top n is the number of top considered contacts, and L is the length of the sequence. "-" means no output for this test case.
Supplementary Data 8b: The precisions of DeepTMP for extracelluar (Extra), cytopliasmic (Cyto), and transmembrane (TM) regions when the AlphaFold2-predicted monomer structures are used as input. Top n is the number of top considered contacts, and L is the length of the sequence. "-" means no output for this test case

File Name: Supplementary Data 9
Description: The max predicted contact probabilities of DeepTMP for 52 transmembrane oligomers from the test set and 50 monomers from the PDBTM database when the experimental monomer structures are used as input. The number of chains of each target is also listed.

File Name: Supplementary Data 10
Description: The PDB IDs of the training, validation, and test sets for the initial training model and the transfer learning model. The PDB IDs of monomer transmembrane proteins are also listed.

File Name: Supplementary Data 11
Description: The links of the protein structures used in this study with the corresponding PDB ID.
