# Supplementary material for: Deep transfer learning for inter-chain contact predictions of transmembrane protein complexes
Source: Nat Commun. 2023 Aug 15;14:4935. doi: 10.1038/s41467-023-40426-3 (PMC10427616; doi:10.1038/s41467-023-40426-3)
Supplement: Supplementary file 5 — Reporting Summary [file 41467_2023_40426_MOESM5_ESM.pdf]

nature portfolio

Corresponding author(s): Sheng-You Huang
Last updated by author(s): Jul 6, 2023

Reporting Summary

Nature Portfolio wishes to improve the reproducibility of the work that we publish. This form provides structure for consistency and transparency in reporting. For further information on Nature Portfolio policies, see our Editorial Policies and the Editorial Policy Checklist.

Statistics

For all statistical analyses, confirm that the following items are present in the figure legend, table legend, main text, or Methods section.

n/a	Confirmed	
☐	☒	The exact sample size (n) for each experimental group/condition, given as a discrete number and unit of measurement
☒	☐	A statement on whether measurements were taken from distinct samples or whether the same sample was measured repeatedly
☒	☐	The statistical test(s) used AND whether they are one- or two-sided<br>*Only common tests should be described solely by name; describe more complex techniques in the Methods section.*
☒	☐	A description of all covariates tested
☒	☐	A description of any assumptions or corrections, such as tests of normality and adjustment for multiple comparisons
☐	☒	A full description of the statistical parameters including central tendency (e.g. means) or other basic estimates (e.g. regression coefficient) AND variation (e.g. standard deviation) or associated estimates of uncertainty (e.g. confidence intervals)
☒	☐	For null hypothesis testing, the test statistic (e.g. F, t, r) with confidence intervals, effect sizes, degrees of freedom and P value noted<br>*Give P values as exact values whenever suitable.*
☒	☐	For Bayesian analysis, information on the choice of priors and Markov chain Monte Carlo settings
☒	☐	For hierarchical and complex designs, identification of the appropriate level for tests and full reporting of outcomes
☒	☐	Estimates of effect sizes (e.g. Cohen's d, Pearson's r), indicating how they were calculated

Our web collection on statistics for biologists contains articles on many of the points above.

Software and code

Policy information about availability of computer code

Data collection	MMseq2 (https://github.com/soedinglab/MMseqs) for sequence cluster and removing the redundant sequence;<br>HHblits (V3.0.3, https://github.com/soedinglab/hh-suite)for searching the multiple sequence alignment;<br>CCMPred (https://github.com/soedinglab/CCMpred) for calculating the DCA;<br>ESM (https://github.com/facebookresearch/esm) for generating the sequence representation and attention matrix;<br>PDBTM (http://pdbtm.enzim.hu/) for collecting the transmembrane protein complexes;<br>AlphaFold2 (v2.0.0, https://github.com/deepmind/alphafold) for generating the monomer structures;<br>AlphaFold-Multimer (v2.2.0, https://github.com/deepmind/alphafold) for generating the complexes.
Data analysis	Chimera (version 1.14, https://www.cgl.ucsf.edu/chimera/) for data visualization;<br>MM-align (Version 20191021; https://zhanggroup.org/MM-align);<br>DeepTMP (http://huanglab.phys.hust.edu.cn/DeepTMP) developed in this study;<br>Python (version 3.7.0, https://www.python.org/);<br>PyTorch(version 1.8.0+cu112, https://pytorch.org/).

For manuscripts utilizing custom algorithms or software that are central to the research but not yet described in published literature, software must be made available to editors and reviewers. We strongly encourage code deposition in a community repository (e.g. GitHub). See the Nature Portfolio guidelines for submitting code & software for further information.

Data

Policy information about availability of data

All manuscripts must include a data availability statement. This statement should provide the following information, where applicable:

- Accession codes, unique identifiers, or web links for publicly available datasets
- A description of any restrictions on data availability
- For clinical datasets or third party data, please ensure that the statement adheres to our policy

All the source data are provided in the Supplementary Data with this paper. The used protein structures in this study are all available in the PDB (https://www.rcsb.org/) and PDBTM databases (http://pdbtm.enzim.hu/). A full list with links of soluble and transmembrane protein used in this study is available in Supplementary Data 11. The sequence database of Uniref30_2020_03 used in this study is available at https://www.uniprot.org/help/uniref/. The sequence database of Big Fantastic Database (BFD) used in this study is available at https://bfd.mmseqs.com/. Other data that support the findings of this study are available from the corresponding author upon request. The Source Data underlying Figs. 2, 3, 4a, b, 5, 6, 7, Table 1 and Supplementary Tables 1, 2, 3 are provided as Source Data file.

Research involving human participants, their data, or biological material

Policy information about studies with human participants or human data. See also policy information about sex, gender (identity/presentation), and sexual orientation and race, ethnicity and racism.

Reporting on sex and gender	N/A
Reporting on race, ethnicity, or other socially relevant groupings	N/A
Population characteristics	N/A
Recruitment	N/A
Ethics oversight	N/A

Note that full information on the approval of the study protocol must also be provided in the manuscript.

Field-specific reporting

Please select the one below that is the best fit for your research. If you are not sure, read the appropriate sections before making your selection.

☒ Life sciences ☐ Behavioural & social sciences ☐ Ecological, evolutionary & environmental sciences

For a reference copy of the document with all sections, see nature.com/documents/nr-reporting-summary-flat.pdf

Life sciences study design

All studies must disclose on these points even when the disclosure is negative.

Sample size	The dataset for transfer learning method was obtained from the PDBTM database (http://pdbtm.enzim.hu/). We collected all the homo-oligomers deposited on PDBTM before January 2021. The screened 2020 transmembrane protein complexes were filtered with the following criteria: (i) The chain number of complexes is equal to the order of symmetry; (ii) Any pair of chains in the assembly share more 99% sequence identity. The final 1907 complexes were clustered by MMseqs2 with a sequence identity cutoff of 30% which resulted in 322 clusters. For each cluster, the complexes with best resolution was chosen as representative. Due to the limitation of ESM-MSA-1b, we excluded the complexes with monomer sequence length of > 1024. Thus, we had a dataset of 309 complexes which were randomly divided into 185, 62 and 62 structures for training, validation and test set. For the 62 targets of test sets, we used the following criteria for filtering. (i) The maximum area of the interface between any pair of chains in the complex is < 500 Å2; (ii) The number of contacts in the interface is < 10. Therefore, there are remained 52 targets in the test sets (Supplementary Data 10).<br>The training dataset for the initial model was obtained from DeepHomo (Yan, Y. and Huang, S.-Y. Accurate prediction of inter-protein residue-residue contacts for homo-oligomeric protein complexes. Briefings in bioinformatics 2021;22(5):bbab038). The datasets including 4132 non-redundant homodimeric complexes structure which are divided into 3532, 300, 300 for training, validation and test sets. Then, we used MMSeqs2 with an E-value of 0.1 to remove the redundant sequence from the training and validation sets of homodimers for initial model, which results in 3079 and 271 targets in the two sets, respectively (Supplementary Data 10). We also used an E-value of 0.1 to remove the redundant sequence between the training/validation sets and the test sets of transmembrane protein complexes. As results, the training and valid sets have remained with 100 and 38 transmembrane homo-oligomers (Supplementary Data 10).
Data exclusions	We use MMseqs2 to filter out redundant protein sequence with >30% identity in the dataset of transmembrane protein complexes. And we also used MMseqs2 with E-value of 0.1 to remove the redundant sequence of training/valid of transmembrane protein complexes. In addition, the complexes with monomer sequence length larger than 1024 are removed because the limitation of ESM-MSA-1b model.

Replication	All results could be reproduced by the download package of DeepTMP with the same input (i.e, the same multiple sequence alignment and monomer tertiary structure)
Randomization	The datasets of DeepTMP and their deposited PDB structures were collected from the PDB and PDBTM database. The dataset are randomly divided into training, validation and test sets for initial training and transfer learning with rational ratio, after the consideration of non-redundancy within each set and among training, validation and test sets.
Blinding	The datasets for initial training model were downloaded from Protein Data Bank (PDB) and the datasets for transfer learning model were downloaded from PDBTM database. Both databases are public database. The redundant sequence of training/validation datasets are removed according to a stringent E-value of 0.1. Therefore, the test dataset can also be considered a blind test dataset that has no overlap with the training/validation datasets.

Reporting for specific materials, systems and methods

We require information from authors about some types of materials, experimental systems and methods used in many studies. Here, indicate whether each material, system or method listed is relevant to your study. If you are not sure if a list item applies to your research, read the appropriate section before selecting a response.

Materials & experimental systems

n/a	Involved in the study
☒	☐ Antibodies
☒	☐ Eukaryotic cell lines
☒	☐ Palaeontology and archaeology
☒	☐ Animals and other organisms
☒	☐ Clinical data
☒	☐ Dual use research of concern
☒	☐ Plants

Methods

n/a	Involved in the study
☒	☐ ChIP-seq
☒	☐ Flow cytometry
☒	☐ MRI-based neuroimaging
